# The nutritional profile of commercial complementary foods in Japan: comparison between low- and high-price products

**DOI:** 10.1017/S0007114523000612

**Published:** 2023-11-14

**Authors:** Minami Sugimoto, Xiaoyi Yuan, Ken Uechi, Satoshi Sasaki

**Affiliations:** 1 Institute for Future Initiatives, University of Tokyo, Tokyo 113-0033, Japan; 2 Department of Environmental and Occupational Health, School of Medicine, Toho University, Tokyo 143-8540, Japan; 3 Department of Nutritional Epidemiology and Shokuiku, National Institute of Biomedical Innovation, Health and Nutrition, Tokyo 162-8636, Japan; 4 Division of Community Health Nursing, Faculty of Health Science, Toho University, 2-2-1 Miyama, Chiba 274-8510, Japan; 5 Department of Social and Preventive Epidemiology, School of Public Health, University of Tokyo, Tokyo 113-0033, Japan

**Keywords:** Baby food, Infant, Young children, Nutritional profile, Food additives, Micronutrients, Ingredients

## Abstract

Despite the increasing market share of commercial complementary foods, their nutritional characteristics and those associated with the price of products are still unknown in Japan. We compared the nutritional characteristics of commercially available complementary foods of different price levels in Japan. Data were obtained from the websites of Japanese brands of infant and young children’s food. Nutrient profiles (unit/100 g), ingredients and food additives were compared between low- and high-priced products by product type. Sixty-three dry meals, 425 soft meals, 187 snacks and sweets, and 60 drinks were analysed. One-fifth of meals and snacks exceeded the CODEX-defined limit (200 mg Na/100 g). Most products lacked content information on nutrients non-mandated to be indicated. High-priced soft meals contained more protein (2·5 *v*. 1·9 g/100 g) and less Na (0·18 *v*. 0·46 g/100 g), less frequently used ≥ 1 added sugar (23 % *v*. 82 %), and less frequently used food additives than low-priced products; however, they had a lower variety of ingredients. The prevalence of products containing ≥ 1 added sugar was higher in low-priced snacks and sweets (91 % *v*. 77 %) but lower in drinks (48 % *v*. 84 %) than in their high-priced counterparts. High Na content is a concern among commercial complementary foods in Japan. Nonetheless, the relationship between the price and nutritional profile of these foods differs by product type. High-priced soft meals might be more favourable regarding nutrient content but not the variety of ingredients than low-priced counterparts. These findings elucidate the nutritional characteristics of commercial complementary foods in Japan.

Early-life dietary habits influence future food preferences and dietary intake^(1–4)^. In the last decade, market sales and use of commercial complementary foods have increased substantially worldwide^(5–7)^. Consequently, commercial complementary foods play a vital role in the dietary intake of infants and young children. Thus, it is essential to describe the products’ nutritional characteristics for better nutrition policies and practices for infants and young children.

Previous studies have reported nutritional concerns regarding the content of commercial complementary foods. For example, high levels of Na and sugar and the presence of sweet foods have been reported in studies from the UK^(8)^, the USA^(9,10)^, Australia^(11,12)^, European countries^(5,13–16)^, Taiwan^(17)^ and South-East Asian countries^(18)^. Other studies have reported a low variety of vegetables and other ingredients in commercial complementary foods^(19–21)^. These studies were mainly published in Western countries. Since complementary foods differ by country^(5,18)^, their nutritional characteristics should be monitored and described in each country.

In addition to nutritional characteristics, the price of products is a major food-choice-related motive for consumers^(22)^. Food and diet costs could contribute to socio-economic disparities in diet quality^(23)^. Previous studies have examined whether healthy products are more expensive than general foods and drinks^(24,25)^. However, none have examined the association between the prices of products and their nutritional characteristics in commercial complementary foods. As commercial complementary foods are convenient food choices for parents with young children aged < 3 years, it is essential to examine the nutritional characteristics of products for young children in association with product price and nutrition.

Few studies have examined the characteristics of commercial complementary foods in Asian countries, including Japan. Further, the relationship of the characteristics of commercial complementary foods with price have rarely been examined. The current Japanese diet is characterised by high Na intake and low intake of dietary fibre, Ca, saturated fat and free sugars^(26–28)^. Among Japanese toddlers aged 18–35 months, free sugar intake is also relatively low: the mean free sugar intake was 17·4 g/d (6·1 % of energy) for boys and 18·2 g/d (6·9 % of energy)^(27)^. Dietary intake among Japanese children may reflect the dietary habits of adults. It is possible that the characteristics of commercial complementary foods are similar to the Japanese dietary characteristics described above.

Disparities in diet quality related to different socio-economic statuses have seldom been reported in Japan. A previous study conducted among children aged 6–18 years showed that such disparities may be reduced by the school lunch programmes in elementary and junior high schools^(29)^. As similar lunch-providing systems are also available in many Japanese nursery schools, protective effects against socio-economic status differences in diet quality may also be expected in pre-schoolers^(30)^. School lunches at nursery were usually prepared from scratch at school or outside cooking facilities^(31)^. For children aged < 3 years, however, diet quality may be more sensitive to monetary costs due to a lower dependence on the lunch programme. For example, 38·1 % in 2015 and 50·4 % in 2020 for children < 3 years old attending nursery facilities^(32)^ and the others (i.e. 61·9 % in 2015 and 49·6 % in 2020) were cared at home by parent(s). In Japan, market sales of commercial complementary foods have been increasing despite a decrease in the birth rate^(7)^. This increasing sales might be related the increasing in female employment rate in Japan^(33)^. Some parents might frequently use commercial complementary foods for convenience, because one-third of mothers think preparing complementary foods is burdensome^(34)^. Although the intake amount of commercial complementary foods among Japanese infants and young children is unknown, it is possible that their contribution to children’s diets has been increasing. The national survey of infants in 2007 reported that 28·0 % and 47·8 % of parents answered ‘frequently’ and ‘sometimes’ used baby food, respectively^(35)^. Considering the increase in the sale of commercially complementary food in Japan in the last decade, the frequency of baby food usage among parents might be higher than in 2007. Thus, this study aimed to (i) describe the information provided by commercially available complementary foods for infants and young children in Japan, focusing on nutrient profiles, ingredients, and food additives and (ii) compare these contents between the price levels of products.

## Methods

### Data collection

Data were primarily obtained from the websites of fifty-four Japanese brands for infant and young children’s food between March and July 2022. The products were first chosen from five leading manufacturers (Wakodo, Asahi Group Foods, Ltd; Kewpie Corporation; Pigeon Corporation; Bean Stalk Snow. Co. Ltd; and Glico Co., Ltd), which produce infant and toddler foods in Japan and participated in *Nihon Baby Food Kyogikai* (an organisation run by companies that manufacture baby food in Japan)^(36,37)^. Although the exact percentage of these companies’ market share is unknown, products from the five leading companies almost dominate (i.e. estimated to be > 80 %) the market of commercial complementary meal, snack and sweets, and drink^(38)^. Additionally, products sold in food grocery markets, drug stores, retail stores in Tokyo or online stores were all selected. This study included products targeting infants and young children (up to 24 months of age). Products for children older than 24 months were excluded because few products were sold with distinct claims representing the recommended age of ≥ 24 months. Further, baby milk formula was excluded. Products not presenting the recommended age were excluded to distinguish them from other foods not targeting infants and toddlers.

The following information was obtained from the websites: brand name, product name, content weight, nutrient information (per 100 g or serving size), ingredients, food additives, reported serving size, recommended age and price. The nutrient information for energy (kcal), protein (g), total fat (g), carbohydrate (g) and Na (g) were obtained because these were to be mandatorily indicated on the food package in Japan^(39)^. Information on other nutrients, such as Ca, Fe and vitamins, was obtained if available. The price data from online retail or grocery stores were extrapolated. If *Akachan-honpo* (a large retail store providing baby foods and goods) has the price of the target products, that value was used, because the online store of *Akachan-honpo* had the largest variety of products among the surveyed stores. Otherwise, the average price of the other grocery stores was used (namely, AEON, SEIYU, LIFE, SUNDRUG, Matsumotokiyoshi, Yodobashi Camera, Toysrus, or else) if the store provided the products. When the price data were unavailable from retail store but available from manufacturers’ websites, those from manufacturers’ websites were used. Data were obtained between March and July 2022. Ethical approval from an institutional review board was not required for this study because no human participants were involved.

### Product categories

Products were classified according to the category defined in Supplementary Table 1 by the first author (M.S.) based on the product name, description of the package or website, and ingredients. The second author (X.Y.) checked the categorisation, and any disagreement was resolved by discussion between the two authors (M.S. and X.Y.). The category used in the WHO report^(40)^ was modified to adapt to Japanese baby foods. The ‘soft–wet spoonable, ready-to-eat foods’ and ‘meals with chunky pieces’ categories in the 2019 WHO report were merged into ‘soft meals’, which had three subcategories: ‘cereal-based meal’, ‘meat, fish, cheese, pulses or vegetable-based meal, with rice or noodles’, and ‘meat, fish or cheese or vegetable-based meal without rice or noodles’. New categories were generated for products to which the 2019 WHO report categorisation was inapplicable, such as ‘dry, powdered and instant vegetable/fish/meat’, ‘other non-formula drinks’, ‘tea’, ‘meal accompaniments’, ‘sauce’, ‘soup’ and ‘soup stock powder’. Products were categorised into five major categories (i.e. dry meals, soft meals, snacks and sweets, drinks, and others) and further categorised into subcategories. The recommended age was categorised into six groups based on the label of the products: < 5 months (including products labelled ‘[for infants] from 0 [1, 2, 3, or 4] months,’), 5–6 months (‘from 5 [or 6] months’), 7–8 months (‘from 7 [or 8] months’), 9–11 months (‘from 9 [10, or 11] months’), 12 months (‘from 12 months’), and 1 year 4–6 months (‘[for toddlers] from 1 years and 4 [or 6] months’)

### Nutritional profile

The energy density was calculated per 100 g of content. Nutrient densities for protein, fat, carbohydrate and Na were also calculated per 100 g content. Based on the recommendation in CODEX^(41)^, foods containing Na > 200 mg/100 g are considered high-Na foods.

The added sugar content weight (g) is not currently required to be listed in Japan’s food packages, while providing used sugar names (e.g. sugar, glucose, corn syrup, etc.) in the ingredient list is mandated^(39)^. WHO report proposed ‘no added sugar or sweetening agent’ used in commercially available complementary foods for infants and young children between 6 and 36 months^(40)^. Thus, foods containing one or more sources of added sugars were identified from the ingredient list, and their percentage of them was described as an indicator of added sugar. The following ingredients were considered as added sugar based on the WHO report^(40)^: sugar or sucrose, dextrose, fructose, glucose, maltose, galactose, trehalose, (any) syrup, honey, malt extract/malted barley, molasses, and juice (other than lemon or lime juice, as they are not sweet-tasting).

We did not use the nutrient profile model to describe the nutritional characteristics because there is no model applicable to Japanese products. There is no such model defined by the Japanese government for food products for infants and young children. In addition, other profile models such as the Pan American Health Organization Nutrient Profile Model^(42)^ could not be applicable because many products did not show the content amount for some nutrients (including free sugar, saturated fat and *trans*-fatty acid) used in the existing nutrient profile model.

### Food ingredients

The ingredients used were categorised into nineteen food groups: cereals, potatoes, starches, green and yellow vegetables, other vegetables, pulses, mushrooms, seaweed, fruits, eggs, meat, fish and seafood, dairy products, fat and oils, spices, seasonings, soup stocks, sugars and sweeteners, and miscellaneous. Green and yellow vegetables were defined by the Ministry of Labor, Health and Welfare^(43)^, including carrots, spinach, reek, tomatoes, green peppers, garland chrysanthemum (*shungiku*), *mizuna*, chives, Japanese mustard spinach (*komatsuna*), *mitsuba*, bok choy, broccoli, asparagus, pumpkin, green beans, peas, okra, kale and moroheiya. Other vegetables were classified as ‘other vegetables’. Furthermore, miscellaneous groups included sesame seeds, wine and *mirin* (i.e. rice-based alcohol for cooking) used for sauce, vitamins or minerals for fortification, lactic acid bacteria, protein hydrolysate, edible eggshell powder, and citric acid.

### Analysed products

**Fig. 1.**
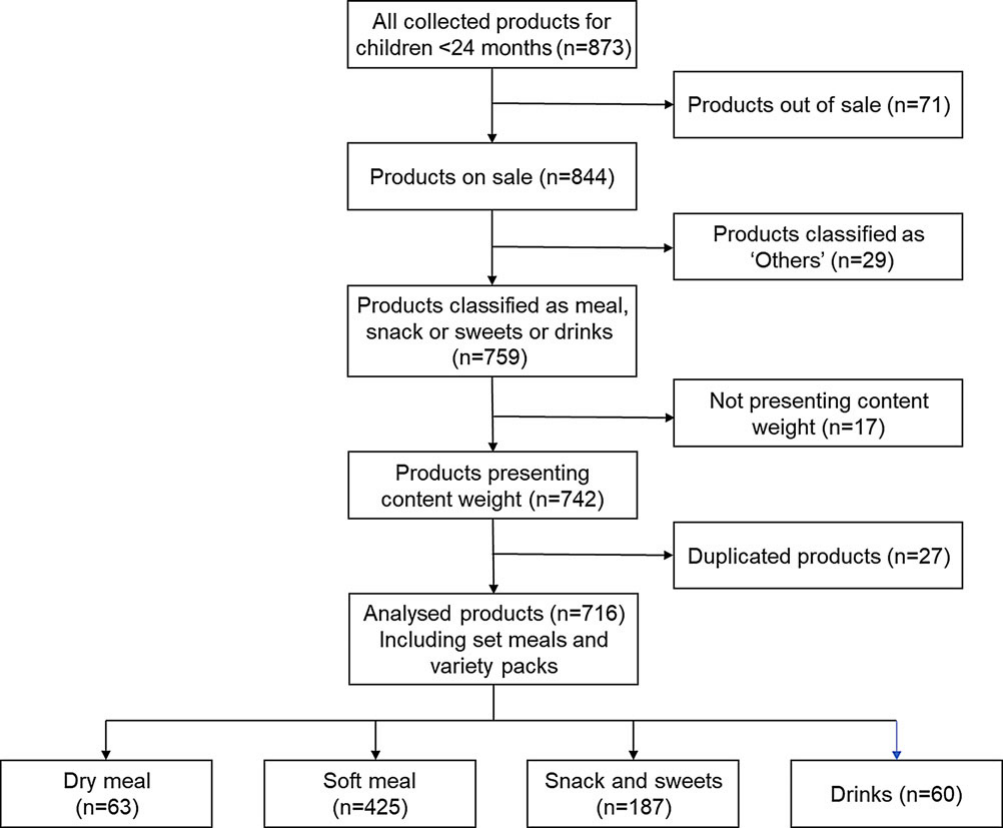
Selection of analysed products. Set meal is consisting of two separate meals: one mainly cereal and the other meat/fish and vegetables.

Of the 873 products obtained, discontinued products during the data collection period (*n* 71) were excluded because the information was no longer available on the manufacturer’s website (Fig. 1). Next, products categorised into ‘others’ (*n* 29) and those that did not provide content weight were excluded from the analysis (*n* 17). Furthermore, products with volume packs or duplicates of other products were excluded from the analysis (*n* 27). Additionally, meals, snacks and sweets, and drinks from a variety packs or set meals (consisting of two separate meals: one mainly cereal and the other meat/fish and vegetables) were excluded from the analysis if they were duplicates of those in other products, for example, for the set meal ‘fish and rice lunch box’ consisting of two meals, ‘rice with vegetable and fish’ and ‘simmered potatoes and pork’, ‘simmered potatoes and pork’ was included in the analysis, while ‘rice with vegetables and fish’ was excluded because the same meal was also included in another set meal.

In total, 60 dry meals, 425 soft meals, 187 snacks and sweets, and 60 drinks from 725 products (including 41 set meals and 14 variety packs) of 49 brands were included in the analysis. Among them, forty-five brands were Japanese domestic manufacturers. Only twenty products were imported from five foreign manufacturers, namely baby bio (France), kiwigarden (New Zealand), Frucht bar (Germany) and GL BIO (Korea).

### Data analysis

All statistical analyses were performed using the SAS statistical software (version 9.4; SAS Institute Inc.). All reported *P*-values were two-tailed. To estimate the effect size, Cohen’s *d* and phi (ϕ) were determined for continuous variables and for categorical variables, respectively. Cohen’s *d* and phi of 0·2, 0·5 and 0·8 were considered as a ‘small’, ‘medium’ and ‘large’ effect size, respectively^(44,45)^. The results were interpreted based on *P*-values and absolute value of Cohen’s *d* or *ϕ*, avoiding the use of arbitrary *P*-value cut-off^(46)^.

The mean, standard deviation (sd), median, and 25 and 75 percentiles of the product price (yen/100 g) were described. Products were dichotomised by price (Japanese yen/100 g): separately by product-type subcategory and then combined by product-type category for analysis. The median value of the price of products at the product-type subcategory level was used as the tentative cut-off.

A *t* test was used to examine the differences in content weight and nutrient content between low- and high-priced products. The *χ* ^2^ test was used to evaluate differences in the prevalence of products containing Na > 200 mg/100 g^(41)^, using ingredients from each food group, containing at least one added sugar, providing information on nutrients non-mandated to be indicated on the package, and containing food additives. The ingredients used were listed by food group and product category. Usage of sweet-taste vegetables was focused because manufacture may use it for palatability and could label ‘vegetable’ on the package for marketing strategy.

## Results

Analysed products primarily came from Wakodo (32 %), followed by Kewpie (12 %), Kindest (11 %) and Pigeon (10 %). Dry meals were mainly targeted at the age groups 5–6 months and 7–8 months (online Supplementary Table 2), while soft meals were mainly targeted at the age groups 7–8 months, 9–11 months and 12 months. The targeted age groups of snacks and sweets differed by subcategories, namely 85 % of ‘confectionery, sweet spreads, and fruit chews’ were mainly used for children aged 12+ months, 100 % of ‘fruit (fresh or dry whole fruit or pieces)’ for 9–11 months and 12 months, 83 % of ‘fruit purée with or without the addition of vegetables, cereals or milk’ for 5–6 months, and 90 % of ‘other snacks and finger foods’ for 7–8 months, 9–11 months, and 12 months. Among drinks, ‘single or mixed fruit juices, vegetable juices’ were targeted at children at 12 months, while ‘other non-formula drinks’ and ‘tea’ were targeted at < 5 months and 5–6 months.

The mean price of products (yen/100 g) was 1290 (sd 1895) for dry meals, 353 (sd 370) for soft meals, 648 (sd 719) for snacks and sweets, and 97 (sd 84) for drinks (Table 1). The mean price of high-priced products was approximately three times that of low-priced products for any food type.

**Table 1.** Price (yen/100 g) of commercial complementary foods sold in Japan for infants and young children (< 24 months) by food type

Food category	Subcategory	All					Low-priced products					High-priced products				
		*n*	Mean	sd	Median	25P, 75P	*n*	Mean	sd	Median	25P, 75P	*n*	Mean	sd	Median	25P, 75P
Dry meals		63	1290	1895	382	270, 1272	33	536	420	270	248, 997	30	2120	2474	810	369, 5000
	Dry, powdered and instant cereal/starchy food	42	525	848	270	257, 382	22	254	20	262	246, 270	20	823	1171	427	355, 810
	Dry, powdered and instant vegetable/fish/meat	22	2821	2443	1272	1170, 5417	11	1100	195	1170	997, 1272	10	4715	2369	5663	1463, 6190
Soft meals		425	352	369	188	149, 410	212	145	25	149	131, 156	213	558	432	410	212, 700
	Cereal-based meal	109	299	180	212	168, 410	52	155	34	156	131, 181	57	430	157	410	349, 500
	Meat, fish, cheese, pulses, or vegetable-based meal, with rice or noodles	89	229	152	152	131, 225	48	138	13	134	131, 152	41	335	171	264	200, 497
	Meat, fish, cheese, pulse or vegetable-based meal without rice or noodles	227	426	467	188	149, 484	111	144	24	148	128, 152	115	701	527	484	212, 1296
Snacks and sweets		187	648	719	367	280, 764	92	284	92	291	231, 343	95	1000	871	764	495, 1061
	Confectionery, sweet spreads and fruit chews	26	701	940	291	186, 484	13	176	67	186	168, 231	13	1226	1114	484	484, 1230
	Fruit (fresh or dry whole fruit or pieces)	9	1920	2109	270	270, 3086	5	242	63	270	270, 270	4	4018	1137	3793	3086, 4950
	Fruit purée with or without the addition of vegetables, cereals or milk	24	270	46	280	239, 311	10	227	38	239	186, 270	14	301	14	311	280, 311
	Other snacks and finger foods	128	618	399	477	331, 854	64	319	81	331	281, 367	64	918	362	854	629, 1027
Drinks		60	97	84	57	22, 179	29	43	27	22	20, 66	31	147	88	160	57, 229
	Single or mixed fruit juices, vegetable juices	29	150	76	160	71, 226	13	71	16	71	57, 75	16	215	27	226	226, 229
	Cow’s milk and milk alternatives with added sugar or sweetening agent	1	34	–	34	34, 34	0	–	–	–	0, 0	1	34	–	34	34, 34
	Other non-formula drinks	11	26	11	22	20, 26	6	21	1	20	0, 0	5	31	14	26	26, 27
	Tea	19	60	67	22	20, 71	10	21	1	21	20, 22	9	104	78	71	57, 108

Tables 2 and 3 show the nutrient content of the products and ingredients used in each product category per 100 g by food price groups. The prevalence of products exceeding 200 mg/100 g Na was 17 % for dry meals, 20 % for soft meals, 41 % for snacks and sweets, and 2 % for drinks. The prevalence of products containing ≥ 1 added sugar was 16 % for dry meals, 52 % for soft meals, 84 % for snacks and sweets, and 74 % for drinks. In dry meals, low-priced products contained less starch; however, there was no significant difference in energy and nutrient content compared with those of high-priced products. Low-priced soft meals had less protein and more Na content than high-priced products; nonetheless, they contained less seaweeds and more green and yellow vegetables, other vegetables, meat, fat and oils, spices, seasonings, soup stocks, and sugars and sweeteners than high-priced products. The prevalence of products containing ≥ 1 added sugar was higher in low-priced soft meals (82 %) than in high-priced ones (23 %). Low-priced snacks and sweets had lower protein and Na content than high-priced products. In addition, they contained eggs more frequently. The prevalence of products containing ≥ 1 added sugar was higher in low-priced snacks and sweets (91 %) than in high-priced ones (77 %), although the prevalence was relatively high in both groups. Similarly, low-priced drinks contained less protein. The prevalence of products containing ≥ 1 added sugar was higher in high- (90 %) than in low-priced drinks (48 %).

**Table 2.** Comparison of content weight, nutrient profile, and ingredients between low- and high-priced (based on price per 100 g) commercial complementary foods (dry meals and soft meals) sold in Japan for infants and young children (< 24 months)*

	Dry meals								Soft meals							
	All		Low-priced products (*n* 33)		High-priced products (*n* 30)			Effect size‡	All		Low-priced products (*n* 212)		High-priced products (*n* 213)			Effect size‡
	Mean	sd	Mean	sd	Mean	sd	*P* †	Cohen’s *d*	Mean	sd	Mean	sd	Mean	sd	*P* †	Cohen’s *d*
Content weight (g)	68·0	46·4	89·1	44·3	44·7	37·0	< 0·001	1·08	106·8	50·2	121·5	62·3	92·2	27·6	< 0·001	0·36
Energy and nutrient profile																
Energy (kcal/100 g)	374	48	373	63	376	21	0·80	0·06	55	20	55	15	55	24	0·85	0·02
Protein (g/100 g)	10·5	5·6	10·4	4·2	10·7	7·0	0·79	0·07	2·2	2·0	1·9	0·8	2·5	2·6	0·002	0·31
Fat (g/100 g)	2·9	4·7	3·4	6·3	2·3	1·7	0·35	0·24	0·9	1·1	0·92	0·86	0·86	1·36	0·59	0·05
Carbohydrate (g/100 g)	78·8	12·2	78·5	14·5	79·3	9·2	0·79	0·07	9·6	3·5	9·7	2·8	9·5	4·1	0·49	0·07
Na (g salt/100 g)	0·49	1·13	0·35	1·12	0·64	1·14	0·32	0·25	0·3	0·3	0·46	0·22	0·18	0·20	< 0·001	1·33
	*n*	%	*n*	%	*n*	%		ϕ	*n*	%	*n*	%	*n*	%		ϕ
Number of products containing > 200 mg/100 g of Na§	11	17	4	12	7	23	0·24	0·15	87	20	77	36	10	5	< 0·001	–0·39
Number of products containing ≥ 1 food item(s) as ingredients from the food group below																
Cereals	45	71	27	82	18	60	0·06	–0·24	233	55	125	59	108	51	0·09	–0·08
Potatoes	15	24	8	24	7	23	0·93	–0·01	186	44	104	49	82	38	0·03	–0·11
Starches	22	35	6	18	16	53	0·004	0·37	206	48	101	48	105	49	0·73	0·02
Green and yellow vegetables||	38	60	19	58	19	63	0·64	0·06	343	81	193	91	150	70	< 0·001	–0·26
Other vegetables	20	32	10	30	10	33	0·80	0·03	329	77	188	89	141	66	< 0·001	–0·27
Pulses	1	2	0	0	1	3	na	0·13	96	23	64	30	32	15	< 0·001	–0·18
Mushrooms	1	2	0	0	1	3	na	0·13	108	25	69	33	39	18	0·001	–0·16
Seaweed	7	11	4	12	3	10	na	–0·03	94	22	23	11	71	33	< 0·001	0·27
Fruits	2	3	0	0	2	7	na	0·19	39	9	25	12	14	7	0·06	–0·09
Eggs	0	0	0	0	0	0	na	na	38	9	30	14	8	4	< 0·001	–0·18
Meat	4	6	2	6	2	7	na	0·01	197	46	123	58	74	35	< 0·001	–0·23
Fish and seafood	10	16	3	9	7	23	na	0·19	160	38	74	35	86	40	0·24	0·06
Dairy products	2	3	0	0	2	7	na	0·19	42	10	31	15	11	5	0·001	–0·16
Fat and oils	21	33	8	24	13	43	0·11	0·20	141	33	105	50	36	17	< 0·001	–0·35
Spices	4	6	1	3	3	10	na	0·14	167	39	127	60	40	19	< 0·001	–0·42
Seasonings	10	16	3	9	7	23	na	0·19	327	77	204	96	123	58	< 0·001	–0·46
Soup stocks	5	8	0	0	5	17	na	0·31	271	64	189	89	82	38	< 0·001	–0·53
Sugars and sweeteners	10	16	2	6	8	27	na	0·28	221	52	173	82	48	23	< 0·001	–0·59
Miscellaneous¶	5	8	1	3	4	13	na	0·19	29	7	11	5	18	8	0·18	0·06
Number of products containing => 1 added sugar	10	16	2	6	8	27	na	0·28	222	52	173	82	49	23	< 0·001	–0·59
Mono- and disaccharides	9	14	2	6	7	23	na	0·25	220	52	173	82	47	22	< 0·001	–0·60
All syrups, nectars, and honey	1	2	0	0	1	3	na	0·13	7	2	7	3	0	0	na	–0·13
Fruit juices or concentrated/powdered fruit juice	2	3	0	0	2	7	na	0·19	4	1	1	0	3	1	na	0·05
Non-sugar sweeteners	0	0	0	0	0	0	na	na	0	0	0	0	0	0	na	na

na, Not applicable to be tested by the *χ* ^2^ test due to the small number of expected frequencies.

* Products were divided into two groups by price (yen/100 g) separately by product-type subcategory and then combined by product-type categories for analysis. The median value of the price of products at the product-type subcategory level was used as the cut-off.

† *P*-value by the *t* test for continuous variables and *χ* ^2^ test for categorical variables.

‡ Cohen’s *d* and phi of 0·2, 0·5 and 0·8 were considered as a ‘small’, ‘medium’ and ‘large’ effect size, respectively.

§ Cited from the CODEX standard for canned baby foods formed by the WHO and FAO of the UN.

|| ‘Green and yellow vegetables’ include carrots, spinach, reek, tomatoes, green peppers, garland chrysanthemum (shungiku), mizuna, chives, Japanese mustard spinach (komatsuna), mitsuba, bok choy, broccoli, asparagus, pumpkin, green beans, peas, okra, kale and moroheiya.

¶ ‘Miscellaneous’ includes sesame seeds, wine and *mirin* (i.e. rice-based alcohol for cooking) used for sauce, vitamins or minerals for fortification, lactic acid bacteria, protein hydrolyzate, edible eggshell powder, and citric acid.

**Table 3.** Comparison of content weight, nutrient profile, and ingredients between low- and high-priced (based on price per 100 g) commercial complementary foods (snacks and sweets and drinks) sold in Japan for infants and young children (< 24 months)*

	Snacks and sweets								Drinks							
	All		Low-priced products (*n* 92)		High-priced products (*n* 95)			Effect size‡	All		Low-priced products (*n* 29)		High-priced products (*n* 31)			Effect size‡
	Mean	sd	Mean	sd	Mean	sd	*P* †	Cohen’s *d*	Mean	sd	Mean	sd	Mean	sd	*P* †	Cohen’s *d*
Content weight (g)	66·0	66·5	95·6	80·5	37·4	28·2	< 0·001	0·96	274·1	190·6	401	148	156	145	< 0·001	1·67
Energy and nutrient profile																
Energy (kcal/100 g)	344	191	317	203	369	175	0·06	0·27	30·9	23·0	27	23	35	22	0·20	0·33
Protein (g/100 g)	5·2	4·9	4·2	4·4	6·2	5·1	0·005	0·42	0·11	0·20	0·01	0·04	0·20	0·2	< 0·001	1·12
Fat (g/100 g)	6·9	9·2	5·7	8·0	8·0	10·1	0·08	0·26	0·009	0·059	0·016	0·084	0·0027	0·015	0·40	0·21
Carbohydrate (g/100 g)	69·4	62·1	70·6	82·9	68·2	31·1	0·79	0·04	7·6	5·6	6·7	5·8	8·4	5·5	0·24	0·31
Na (g salt/100 g)	0·51	0·55	0·40	0·48	0·61	0·60	0·006	0·40	0·07	0·12	0·05	0·07	0·10	0·16	0·12	0·41
	*n*	%	*n*	%	*n*	%		ϕ	*n*	%	*n*	%	*n*	%		ϕ
Number of products containing > 200 mg/100 g of Na§	76	41	29	32	47	49	0·01	0·18	1	2	0	0	1	3	na	0·13
Number of products containing ≥ 1 food item(s) as ingredients from the food group below																
Cereals	103	55	48	52	55	58	0·43	0·06	7	12	3	10	4	13	na	0·04
Potatoes	17	9	11	12	6	6	0·18	–0·10	6	10	0	0	6	19	na	0·32
Starches	108	58	56	61	52	55	0·40	–0·06	7	12	4	14	3	10	na	–0·06
Green and yellow vegetables||	61	33	34	37	27	28	0·21	–0·09	17	28	6	21	11	35	0·20	0·16
Other vegetables	15	8	8	9	7	7	0·74	–0·02	14	23	2	7	12	39	0·004	0·38
Pulses	18	10	10	11	8	8	0·57	–0·04	1	2	0	0	1	3	na	0·13
Mushrooms	0	0	0	0	0	0	na	0·00	1	2	0	0	1	3	na	0·13
Seaweed	19	10	8	9	11	12	0·51	0·05	9	15	0	0	9	29	na	0·41
Fruits	63	34	33	36	30	32	0·53	–0·05	37	62	18	62	19	61	0·95	–0·01
Eggs	44	24	36	39	8	8	< 0·001	–0·36	0	0	0	0	0	0	na	na
Meat	2	1	2	2	0	0	0·15	–0·11	0	0	0	0	0	0	na	na
Fish and seafood	18	10	5	5	13	14	0·06	0·14	0	0	0	0	0	0	na	na
Dairy products	61	33	38	41	23	24	0·01	–0·18	6	10	1	3	5	16	na	0·21
Fat and oils	81	43	34	37	47	49	0·08	0·13	2	3	1	3	1	3	na	–0·01
Spices	5	3	4	4	1	1	na	–0·10	2	3	0	0	2	6	na	0·18
Seasonings	79	42	29	32	50	53	0·004	0·21	14	23	5	17	9	29	0·004	0·14
Soup stocks	10	5	5	5	5	5	na	0·00	3	5	0	0	3	10	na	0·22
Sugars and sweeteners	144	77	78	85	66	69	0·01	–0·18	40	67	14	48	26	84	na	0·38
Miscellaneous¶	33	18	17	18	16	17	0·77	–0·02	27	45	9	31	18	58	0·04	0·27
Number of products containing => 1 added sugar	157	84	84	91	73	77	0·007	–0·20	40	67	14	48	26	84	0·004	0·38
Mono- and disaccharides	144	77	78	85	66	69	0·01	–0·18	22	37	10	34	12	39	0·73	0·04
All syrups, nectars, and honey	48	26	32	35	16	17	0·005	–0·21	24	40	6	21	18	58	0·003	0·38
Fruit juices or concentrated/powdered fruit juice	28	15	20	22	8	8	0·01	–0·19	5	8	0	0	5	16	na	0·29
Non-sugar sweeteners	7	4	1	1	6	6	na	0·14	5	8	2	7	3	10	na	0·05

na, Not applicable to be tested by *χ* ^2^ test due to the small number of expected frequency.

* Products were divided into two groups by price (yen/100 g) separately by product-type subcategory and then combined by product-type categories for analysis. The median value of the price of products at the product-type subcategory level was used as the cut-off.

† *P*-value by the *t* test for continuous variables and *χ* ^2^ test for categorical variables.

‡ Cohen’s *d* and phi of 0·2, 0·5 and 0·8 were considered as a ‘small’, ‘medium’ and ‘large’ effect size, respectively.

§ Cited from the CODEX standard for canned baby foods formed by the WHO and FAO of the UN.

|| ‘Green and yellow vegetables’ include carrots, spinach, reek, tomatoes, green peppers, garland chrysanthemum (shungiku), mizuna, chives, Japanese mustard spinach (komatsuna), mitsuba, bok choy, broccoli, asparagus, pumpkin, green beans, peas, okra, kale and moroheiya.

¶ ‘Miscellaneous’ includes sesame seeds, wine and *mirin* (i.e. rice-based alcohol for cooking) used for sauce, vitamins or minerals for fortification, lactic acid bacteria, protein hydrolyzate, edible eggshell powder, and citric acid.

In soft meals, 90 % and 69 % of low-priced products contained carrots and onions, respectively (online Supplementary Table 3); nonetheless, these vegetables were not as dominant in high-priced products (48 % and 55 %, respectively) as in low-priced products. The usage frequency of other sweet-tasting vegetables was also higher in low-priced products, namely pumpkin (25 % in low-priced and 15 % in high-priced products), tomato (29 % and 17 %) and sweet corn (34 % and 10 %) than in high-priced products.

Table 4 presents the number of products that provide information on nutrients non-mandated to be indicated on the package. In general, information regarding saturated fat, dietary fibre, and other micronutrients, except for Ca and Fe, was rarely provided in any product category. For dry products, there was no significant difference in the frequency of information provided on Ca and Fe content. In contrast, high-priced soft meals more frequently provided information about vitamin A, Ca and Fe than low-priced products. Similarly, high-priced snacks and sweets more frequently provided information on dietary fibre, Ca and Fe than low-priced products. Drinks rarely provided information regarding nutrients.

**Table 4.** Comparison of the number of products providing content information of nutrients not mandated to show on the package between the low- and high-priced commercial complementary foods sold in Japan for infants and young children*

	Dry meals								Soft meals								Snacks and sweets								Drinks							
	All (*n* 63)		Low-priced product (*n* 33)		High-priced product (*n* 30)		*P*	ϕ†	All (*n* 425)		Low-priced product (*n* 212)		High-priced product (*n* 213)		*P*	ϕ†	All (*n* 187)		Low-priced product (*n* 92)		High-priced product (*n* 95)		*P*	ϕ†	All (*n* 60)		Low-priced product (*n* 29)		High-priced product (*n* 31)		*P*	ϕ†
	*n*	%	*n*	%	*n*	%			*n*	%	*n*	%	*n*	%			*n*	%	*n*	%	*n*	%			*n*	%	*n*	%	*n*	%		
Saturated fat	7	11	5	15	2	7	na	–0·13	0	0	0	0	0	0	na	na	8	4	6	7	2	2	na	–0·11	0	0	0	0	0	0	na	na
Dietary fibre	4	6	2	6	2	7	na	0·01	7	2	0	0	7	3	na	0·13	25	13	5	5	20	21	0·002	0·23	6	10	4	14	2	6	na	–0·12
Vitamin A	2	3	2	6	0	0	na	–0·17	58	14	1	0	57	27	< 0·001	0·38	3	2	0	0	3	3	na	0·13	0	0	0	0	0	0	na	na
Vitamin D	1	2	0	0	1	3	na	0·13	36	8	0	0	36	17	na	0·30	5	3	0	0	5	5	na	0·16	0	0	0	0	0	0	na	na
Vitamin E	1	2	0	0	1	3	na	–0·12	0	0	0	0	0	0	na	na	0	0	0	0	0	0	na	na	0	0	0	0	0	0	na	na
Thiamin	1	2	1	3	0	0	na	–0·12	0	0	0	0	0	0	na	na	2	1	1	1	1	1	na	–0·002	0	0	0	0	0	0	na	na
Riboflavin	1	2	0	0	1	3	na	0·13	0	0	0	0	0	0	na	na	0	0	0	0	0	0	na	na	0	0	0	0	0	0	na	na
Niacin	0	0	0	0	0	0	na	na	0	0	0	0	0	0	na	na	0	0	0	0	0	0	na	na	0	0	0	0	0	0	na	na
Vitamin B_6_	1	2	1	3	0	0	na	–0·12	0	0	0	0	0	0	na	na	0	0	0	0	0	0	na	na	0	0	0	0	0	0	na	na
Vitamin B_12_	0	0	0	0	0	0	na	na	0	0	0	0	0	0	na	na	0	0	0	0	0	0	na	na	0	0	0	0	0	0	na	na
Folate	0	0	0	0	0	0	na	na	5	1	5	2	0	0	na	–0·11	0	0	0	0	0	0	na	na	0	0	0	0	0	0	na	na
Vitamin C	0	0	0	0	0	0	na	na	0	0	0	0	0	0	na	na	0	0	0	0	0	0	na	na	3	5	0	0	3	10	na	–0·24
Potassium	0	0	0	0	0	0	na	na	0	0	0	0	0	0	na	na	0	0	0	0	0	0	na	na	10	17	5	17	5	16	na	–0·01
Ca	15	24	6	18	9	30	na	0·14	129	30	38	18	91	43	< 0·001	0·27	100	53	44	48	56	59	0·13	0·11	5	8	1	3	4	13	na	0·17
Mg	0	0	0	0	0	0	na	na	0	0	0	0	0	0	na	na	0	0	0	0	0	0	na	na	0	0	0	0	0	0	na	na
Zn	0	0	0	0	0	0	na	na	2	0	0	0	2	1	na	0·07	0	0	0	0	0	0	na	na	0	0	0	0	0	0	na	na
Fe	9	14	3	9	6	20	na	0·16	128	30	43	20	85	40	< 0·001	0·21	57	30	22	24	35	37	0·05	0·14	7	12	1	3	6	19	na	0·25

na, Not applicable to be tested by *χ* ^2^ test due to the small number of expected frequencies.

* Products were divided into two groups by price (yen/100 g) separately by product type subcategory and then combined by product-type categories for analysis. The median value of the price of products at the subcategory level was used as a cut-off.

† Cohen’s *d* and phi of 0·2, 0·5 and 0·8 were considered as a ‘small’, ‘medium’ and ‘large’ effect size, respectively.

The prevalences of products containing food additives are shown in Table 5. No food additives were used in more than half of the dry meals (63 %) and in nearly half of the soft meals (42 %). In contrast, more than half of all snacks and sweets (71 %) and drinks (90 %) contained one or more food additives. Low-priced dry meals and high-priced soft meals contained food additives less frequently than their counterparts. Regarding snacks, sweets and drinks, there was little difference in the frequency of food additive usage and types between low- and high-priced products.

**Table 5.** Comparison of food additives between low and high priced (based on price per 100 g) commercial complementary foods sold in Japan for infants and young children (< 24 months)*

	Dry meals								Soft meals								Snacks and sweets								Drinks							
	All (*n* 63)		Low-priced product (*n* 33)		High-priced product (*n* 30)		*P*	ϕ†	All (*n* 425)		Low-priced products (*n* 212)		High-priced products (*n* 213)		*P*	ϕ†	All (*n* 187)		Low-priced product (*n* 92)		High-priced product (*n* 95)		*P*	ϕ†	All (*n* 60)		Low-priced product (*n* 29)		High-priced product (*n* 31)		*P*	ϕ†
	*n*	%	*n*	%	*n*	%			*n*	%	*n*	%	*n*	%			*n*	%	*n*	%	*n*	%			*n*	%	*n*	%	*n*	%		
Number of food additives																																
0	40	63	25	76	15	50	0·01	0·37	179	42	44	21	135	63	< 0·001	0·51	54	29	20	22	34	36	0·04	0·19	6	10	2	7	4	13	na	0·15
1	13	21	7	21	6	20			133	31	113	54	20	9			41	22	26	28	15	16			17	28	10	34	7	23		
≥ 2	10	16	1	3	9	30			113	27	55	26	58	27			92	49	46	50	46	48			37	62	17	59	20	65		
Type of food additives																																
Acidity regulator	3	5	1	3	2	7	na	0·09	7	2	7	3	0	0	na	–0·13	21	11	17	18	4	4	0·002	–0·23	31	52	12	41	19	61	0·04	0·20
Anti-caking agent	1	2	0	0	1	3	na	0·13	0	0	0	0	0	0	na	na	0	0	0	0	0	0	na	na	0	0	0	0	0	0	na	na
Antioxidant	12	19	5	15	7	23	0·41	0·10	3	1	3	1	0	0	na	–0·08	32	17	17	18	15	16	0·63	–0·04	10	17	9	31	1	3	na	–0·37
Coagulant	0	0	0	0	0	0	na	na	25	6	19	9	6	3	0·007	–0·13	0	0	0	0	0	0	na	na	0	0	0	0	0	0	na	na
Colour	2	3	1	3	1	3	na	0·01	0	0	0	0	0	0	na	na	4	2	3	3	1	1	na	–0·08	3	5	1	3	2	6	na	–0·08
Emulsifier	0	0	0	0	0	0	na	na	1	0	0	0	1	0	na	0·05	33	18	16	17	17	18	0·93	0·01	12	20	3	10	9	29	0·07	0·23
Flavorings	0	0	0	0	0	0	na	na	0	0	0	0	0	0	na	na	30	16	16	17	14	15	0·62	–0·04	24	40	13	45	11	35	0·46	–0·10
Flavour enhancer	4	6	1	3	3	10	na	0·14	0	0	0	0	0	0	na	na	1	1	0	0	1	1	na	0·07	6	10	2	7	4	13	na	0·10
Raising agent	0	0	0	0	0	0	na	na	0	0	0	0	0	0	na	na	48	26	25	27	23	24	0·64	–0·03	0	0	0	0	0	0	na	na
Stabiliser	0	0	0	0	0	0	na	na	0	0	0	0	0	0	na	na	2	1	1	1	1	1	na	na	7	12	0	0	7	23	na	0·14
Sweetener	0	0	0	0	0	0	na	na	0	0	0	0	0	0	na	na	1	1	0	0	1	1	na	0·07	2	3	0	0	2	6	na	0·18
Thickener	0	0	0	0	0	0	na	na	172	41	159	75	13	6	< 0·001	–0·70	23	12	10	11	13	14	0·56	0·04	21	35	2	7	19	61	< 0·001	0·57
Vitamins (fortification)	0	0	0	0	0	0	na	na	29	7	2	1	27	13	< 0·001	0·23	9	5	3	3	6	6	na	0·07	13	22	9	31	4	13	0·09	–0·22
Ca (fortification)	11	17	2	6	9	30	0·01	0·31	90	21	34	16	56	26	0·01	0·13	95	51	47	51	48	51	0·94	–0·01	4	7	0	0	4	13	na	0·26
Fe (fortification)	6	10	0	0	6	20	na	0·34	106	25	43	20	63	30	0·03	0·11	57	30	23	25	34	36	0·11	0·12	8	13	1	3	7	23	na	0·28

na, Not applicable to be tested by *χ* ^2^ test due to the small number of expected frequencies.

* Products were divided into two groups by price (yen/100 g) separately by product type subcategory and then combined by product-type categories for analysis. The median value of the price of products at the product-type subcategory level was used as a cut-off.

† Cohen’s *d* and phi of 0·2, 0·5 and 0·8 were considered as a ‘small’, ‘medium’ and ‘large’ effect size, respectively.

## Discussion

To our knowledge, this is the first study to compare the nutrient content, ingredients and food additive usage between the price levels of commercial complementary foods. Additionally, it is the first study to describe the nutritional profile of commercial complementary foods in Japan. The study highlights the potential concerns of a high level of Na in soft meals (> 200 mg/100 g), and the frequency of using ≥ 1 added sugar in soft meals, snacks and sweets, and drinks, although the quantity of added sugar is unknown. Low-priced soft meals had a greater variety of ingredients but less protein, higher Na, and a higher proportion of added sugar than high-price soft meals.

High-priced products had more favourable Na and protein contents for soft meals and snacks and sweets. In addition, high-priced products less frequently used ≥ 1 added sugar and more frequently provided information about vitamin A, Ca, and Fe, less frequently used food additives, and more frequently fortified vitamins and minerals than low-priced products. Although it is unknown whether these high-priced products have on-pack claims about the content of vitamins and minerals, they might use that information as a marketing strategy. Further study is needed to investigate the difference in marketing strategy among products, including on-pack labels and claims among commercial complementary food products in Japan. Surprisingly, high-priced products were over three times more expensive than low-priced products. Large price disparities among products could cause diet disparities between children of different socio-economic statuses^(23)^ due to the relatively higher dependence of diet on caregivers at home^(32)^. However, differences in nutrient profiles between the high- and low-priced groups might be relatively small compared with differences between products in Japan and those in other countries. Additionally, the detailed nutritional profile of meals was unknown due to the limited availability of information for nutrients non-mandated to be indicated. In other countries, content information was provided for saturated fat^(9,11,12,15)^, fibre^(47)^ and sugar^(9–11,15,47,48)^, although it was rarely provided for other minerals and vitamins^(11)^, which is similar to the findings of the present study. It is possible that parents choose products based on package claims and images^(49,50)^ rather than nutritional characteristics due to their poor understanding of ingredients. Some parents might believe that higher-priced products are of better quality. Currently, there is no official recommendation in Japan from the Ministry of Health, Labour, and Welfare on how to read the food label for the population. A previous study reported that only 35·9 % of women with higher educational backgrounds ‘always’ or ‘sometimes’ read food labels while shopping^(51)^. Although there is no study about the frequency of reading labels among Japanese parents to our knowledge, it is not expected that the proportion would be substantially higher than that for women with higher education. Thus, for appropriate food selection among parents, more detailed information on nutrient content and consumer education is required.

Soft meals formed the largest fraction of products included in this study, with the most substantial differences observed in nutrient profiles between price levels. The energy density (55 kcal/100 g) observed in this study was similar to or lower than that observed in previous studies conducted in other countries (60–99 kcal/100 g)^(8,11,12,14,20,47,48,52)^. In addition, soft meals in Japan had similar protein content (2·2 g/100 g) to those in the UK (2·6–3·1 g/100 g)^(8,20)^, Germany (1·4 g/100 g)^(14)^ and South-East Asian countries (1·9–3·2 g/100 g)^(18)^; however, they had lower protein content than soft meals in Australia (2·2–4·4 g/100 g)^(11,12,48)^ and Italy (1·8–6·0 g/100 g)^(15)^. The total fat content in soft meals observed in this study (0·9 g/100 g) was also similar to or lower than that in previous studies^(8,12,14,15,18,20,48)^ (0·5–3·2 g/100 g). The WHO proposed the minimum energy density to be 60 kcal/100 g, minimum protein content, > 3·0 g/100 kcal, and maximum total fat content, 4·5 g /100 kcal for commercially available soft–wet spoonable, ready-to-eat foods^(40)^. Although soft meals have acceptable fat content, improving their energy and protein levels may be required, considering the recommendation by the WHO and the content of products in other countries.

The Na content in commercial complementary foods in Japan is relatively high compared with other countries. A previous study in the USA also reported potential concerns about higher Na content^(9)^: the mean Na content was 30 mg/100 g (0·076 g salt/100 g) in ‘dinners, soups, and vegetables, stages 2 and 3’ for infants, 174 mg/100 g (0·44 g salt/100 g) in ‘dinners or meals’ for toddlers, and 447 mg/100 g (1·13 g salt/100 g) in ‘savoury snacks’. Furthermore, the proportion of products exceeding a Na content of 200 mg/100 g was 0 % for products for infants, 5 % for ‘dinners or meals’ and 69 % for ‘savoury snacks’^(9)^. In other studies, the Na content of soft meals was approximately 10–60 mg/100 g (0·02–0·15 g salt/100 g)^(8,14,15,18,48)^. Although the association between early-life exposure to tastes and taste preference in later life is controversial^(4,53–55)^, it is possible that exposure to salty foods in early life is associated with easy acceptance of salty tastes in later life. Due to the higher Na intake in Japanese adults^(26)^, infants and young children in Japan could be easily exposed to a higher Na diet both at their current age and in the future. The Dietary Reference Intakes for Japanese recommends < 3·0 g/d salt intake for children aged 1–2 years^(56)^. However, the adherence rate to this recommendation in this age group is unknown. Moreover, it is also unknown how commercial complementary food contributes to Na intake in this age group. A more dietary survey targeting infants and toddlers is warranted in Japan.

This study found frequent use of ≥ 1 added sugar in soft meals, snacks and sweets, and drinks. This is consistent with the findings of other studies that showed a higher proportion of snacks and sweets containing ≥ 1 added sugar. However, the proportion of meals with ≥ 1 added sugar in our study (16 % of dry products and 52 % of soft meals) was relatively higher than in other studies^(10,13,18)^. For example, in the USA, the proportion of meals with ≥ 1 added sugar was only 4 % in those for infants and 32 % in those for toddlers^(10)^. Manufacturers might use added sugar as seasoning due to preferences for sweet taste among children^(4)^. These sweet tastes can mask the taste and flavour of individual ingredients. Additionally, exposure to added sugar during infancy might result in higher added sugar intake later in life^(56)^. Although the amount of added sugar is unknown in Japanese commercial complementary foods, manufacturers should avoid using added sugar in complementary foods as much as possible.

Surprisingly, low-priced soft meals contained a greater variety of ingredients than high-priced products. The proportion of products containing at least one food item from a specific food group was higher for low-priced than for high-priced products in thirteen food groups (excluding cereals, starches, fruits, fish and seafood). A previous study showed that a variety of flavours in early life might result in higher dietary quality in childhood^(1)^. Hence, lower-priced soft meals are favourable for food variety. We found that 90 % and 80 % of low-priced soft meals contained carrots and onions as ingredients, respectively. The frequent use of carrots and less use of bitter vegetables were consistently reported in the previous studies in Germany^(14)^, the USA^(19)^ and the UK^(20)^. The carrot use frequency in this study (90 % of soft meals) was much higher than that in other studies from the USA^(19)^ (31·9 %) and the UK^(20)^ (19·4 %). The frequency of using bitter vegetables in this study (15 % for spinach and 15 % for broccoli) was much lower than that of carrots, although it was higher than that in studies from the USA (2·0 % for spinach and 2·4 % for broccoli)^(19)^ and the UK (13·5 % for spinach and 7·3 % for broccoli)^(20)^. The frequent use of sweet-tasting vegetables may reflect the innate preferences of infants for sweet tastes^(4)^. The manufacturers probably mixed several vegetables and used sweet tastes to increase the acceptance of the products^(57)^. Similarly, serving mixed fruits and vegetables in one meal was a common dietary practice by parents in the UK^(57)^. Thus, although providing a variety of products in one meal is practical and convenient, children should be provided with single foods, including vegetables, and repeatedly exposed to the same food during the weaning process to increase food acceptability^(58)^.

In the present study, 37 % of dry meals, 58 % of soft meals, 71 % of snacks and sweets, and 90 % of drinks include one or more food additives. Some of them only include compounds of vitamins, Ca, and Fe as additives, and others contain additives exclusively used in ultra-processed foods, including flavours, colours, emulsifiers, artificial sweeteners and thickeners^(59)^. Thus, some of these products might be categorised as ultra-processed foods. In a previous study, 90·6 % of baby biscuits and rusks, 74·1 % of baby snacks, and 79·2 % of baby savoury meals and dishes were categorised as processed or ultra-processed products in European countries^(16)^. The proportion of ultra-processed foods in snacks might be comparable between the current and previous studies. The association between ultra-processed foods and the nutritional and quality of national diets has been concerned. Further study is needed to investigate those association in infants and young children regarding usage of commercial complementary foods in Japan.

The contribution of commercial complementary foods to dietary intake has been increasing in young children^(5–7)^. For example, a study in five European countries showed that over 75 % of energy intake comes from commercial complementary foods at 4–6 months, which decreases to about 30–40 % at 7–12 months^(5)^. No previous study has shown the contribution of commercial complementary foods to diet among young children in the Japanese population. According to the guidelines for weaning by the Ministry of Health, Labour, and Welfare in Japan, preparing complementary food at home is recommended, but using commercial complementary foods is one of the ways to reduce the burden of preparation among parents^(60)^. Additionally, the guide referred to some concerns about commercial complementary foods; however, their scientific evidence or supporting data were not shown^(60)^. Further studies are needed to examine the intake amount and dietary contribution of commercially available foods and their influence on child development for evidence-based recommendations. A nutrient content database of commercially available food is needed for further studies.

This study has some limitations. First, we used the price per weight to compare the products. Previous studies have shown that using different units (price per energy and serving) can yield different results^(61)^. In addition, the price per unit weight may be difficult to interpret. However, it was difficult to calculate the price per energy and serving using the available data. Some products, such as drinks and vegetable-based meals, have low or no energy content. The serving size was not provided and could change with the children’s age. Therefore, the use of price per weight was more appropriate and feasible for this study. Second, comparing our results to those of other studies is challenging because the product-type categories used in this study were not identical to those used in previous studies. We initially attempted to classify products according to the categories proposed by the WHO^(40)^ for standardisation. However, we had to modify it for application to Japanese products because the WHO categories, which were defined based on the products available in European countries, are not always applicable to products in non-European countries. Moreover, previous studies used their own classification methods. Hence, it is necessary to establish a global standard for the classification of commercial complementary foods and corresponding nutritional criteria for the healthy development of infants and young children. Third, we could not collect some products rarely sold online or only be available in physical stores in other areas than Tokyo. However, we intended to cover commercial complementary foods sold in Japan as much as possible, by including products from five leading manufacturers and those reached out from the physical and online stores. Thus, the representativeness of the sample in this study might be acceptable. Fourth, we used the median price value as the tentative cut-off value to dichotomise the products because there is no clear cut-off to identify high-priced products. It could be possible that some products were misclassified into high-priced products due to small weight content. Lastly, we could not describe the quantity of sugar content. Although we compared the proportion of products containing ≥ 1 added sugar according to the WHO guideline of ‘no added sugar or sweetening agent’^(40)^, the results might differ when comparing added sugar content amount. Some products with only one added sugar may have a higher overall content of added sugar than one with ≥ 2 added sugars. Contrarily, it might not be a concern if a product only contains a tiny amount of added sugar. Thus, the results focusing on the proportion of products with ≥ 1 added sugar without sugar amount should be interpreted with caution.

In conclusion, there are concerns about the high salt content, frequent usage of added sugars and lack of information on micronutrients in commercial infant foods in Japan. There is little difference in energy, fat and carbohydrate content between low- and high-priced commercial complementary foods in Japan. Furthermore, although higher-priced soft meals may have less ingredient variety, they tend to be more nutritionally favourable in terms of protein, salt and added sugars. This study contributes to understanding the nutritional characteristics of commercial complementary foods in Japan. Further studies are needed to examine the contribution of commercial complementary foods to dietary intake among Japanese children and their association with household income.

## Supplementary material

For supplementary material accompanying this paper visit https://doi.org/10.1017/S0007114523000612. click here to view supplementary material
